# Supplementary material for: Meta-Analysis and Gene Set Enrichment Relative to ER Status Reveal Elevated Activity of MYC and E2F in the “Basal” Breast Cancer Subgroup
Source: PLoS One. 2009 Mar 9;4(3):e4710. doi: 10.1371/journal.pone.0004710 (PMC2650420; doi:10.1371/journal.pone.0004710)
Supplement: Dataset S1 — GSEA results from first screen. The file contains a zipped archive of the HTML results of the second GSEA screen. Reports for enriched or depleted datasets may be accessed from the “index.html” link within the my_analysis.GseaPreranked.1219975950625.rpt.zip ZIP archive. Enrichment plots are available for each of the 50 most enriched and depleted gene sets. (13.32 MB ZIP) [file pone.0004710.s001.zip › my_analysis.GseaPreranked.1219975950625.rpt.zip!//CHR16P13.html]

Details for gene set CHR16P13[GSEA]

|  || Dataset | zSco.ERneg.to.ERpos\_collapsed |
| Phenotype | NoPhenotypeAvailable |
| Upregulated in class | na\_neg |
| GeneSet | CHR16P13 |
| Enrichment Score (ES) | -0.47951958 |
| Normalized Enrichment Score (NES) | -2.4084346 |
| Nominal p-value | 0.0 |
| FDR q-value | 0.0 |
| FWER p-Value | 0.0 |
Table: GSEA Results Summary

  

Fig 1: Enrichment plot: CHR16P13      
 Profile of the Running ES Score & Positions of GeneSet Members on the Rank Ordered List

  

| PROBE | GENE SYMBOL | GENE\_TITLE | RANK IN GENE LIST | RANK METRIC SCORE | RUNNING ES | CORE ENRICHMENT || 1 | IL32 |  |  | 125 | 5.701 | 0.0070 | No |
| 2 | CSNK2A2 |  |  | 871 | 3.669 | -0.0390 | No |
| 3 | ABCC1 |  |  | 877 | 3.666 | -0.0288 | No |
| 4 | C16ORF5 |  |  | 1113 | 3.346 | -0.0370 | No |
| 5 | SOCS1 |  |  | 1214 | 3.222 | -0.0352 | No |
| 6 | CIITA |  |  | 1216 | 3.220 | -0.0260 | No |
| 7 | MEFV |  |  | 1245 | 3.193 | -0.0188 | No |
| 8 | C16ORF59 |  |  | 1571 | 2.878 | -0.0352 | No |
| 9 | PKMYT1 |  |  | 1715 | 2.753 | -0.0381 | No |
| 10 | TMC7 |  |  | 2005 | 2.526 | -0.0528 | No |
| 11 | CARHSP1 |  |  | 2492 | 2.203 | -0.0834 | No |
| 12 | MMP25 |  |  | 2540 | 2.179 | -0.0806 | No |
| 13 | PDIA2 |  |  | 2558 | 2.169 | -0.0756 | No |
| 14 | ACSM3 |  |  | 2615 | 2.138 | -0.0737 | No |
| 15 | CLDN6 |  |  | 2777 | 2.053 | -0.0800 | No |
| 16 | MSLN |  |  | 2785 | 2.047 | -0.0746 | No |
| 17 | LOC283824 |  |  | 2800 | 2.040 | -0.0697 | No |
| 18 | LOC339047 |  |  | 3003 | 1.930 | -0.0795 | No |
| 19 | ATF7IP2 |  |  | 3038 | 1.914 | -0.0766 | No |
| 20 | PRSS21 |  |  | 3088 | 1.891 | -0.0748 | No |
| 21 | PRM1 |  |  | 3138 | 1.871 | -0.0731 | No |
| 22 | CCNF |  |  | 3265 | 1.809 | -0.0775 | No |
| 23 | KCTD5 |  |  | 3304 | 1.792 | -0.0752 | No |
| 24 | PAQR4 |  |  | 3434 | 1.736 | -0.0799 | No |
| 25 | RNPS1 |  |  | 3539 | 1.690 | -0.0829 | No |
| 26 | LITAF |  |  | 3643 | 1.642 | -0.0860 | No |
| 27 | ZNF200 |  |  | 3880 | 1.561 | -0.0994 | No |
| 28 | CLDN9 |  |  | 4028 | 1.501 | -0.1063 | No |
| 29 | HBQ1 |  |  | 4327 | 1.380 | -0.1249 | No |
| 30 | MMPL1 |  |  | 4377 | 1.364 | -0.1247 | No |
| 31 | TNFRSF12A |  |  | 4486 | 1.323 | -0.1291 | No |
| 32 | C16ORF42 |  |  | 4612 | 1.282 | -0.1349 | No |
| 33 | RPS2 |  |  | 4764 | 1.232 | -0.1428 | No |
| 34 | SNN |  |  | 4809 | 1.220 | -0.1426 | No |
| 35 | PPL |  |  | 4898 | 1.189 | -0.1459 | No |
| 36 | MAPK8IP3 |  |  | 4916 | 1.185 | -0.1437 | No |
| 37 | NTN2L |  |  | 5164 | 1.093 | -0.1594 | No |
| 38 | GFER |  |  | 5279 | 1.055 | -0.1650 | No |
| 39 | TNFRSF17 |  |  | 5616 | 0.939 | -0.1878 | No |
| 40 | AMDHD2 |  |  | 5654 | 0.928 | -0.1879 | No |
| 41 | ZG16 |  |  | 5790 | 0.881 | -0.1956 | No |
| 42 | AXIN1 |  |  | 5865 | 0.855 | -0.1988 | No |
| 43 | ZNF434 |  |  | 5952 | 0.831 | -0.2029 | No |
| 44 | GRIN2A |  |  | 6104 | 0.781 | -0.2121 | No |
| 45 | RPL3L |  |  | 6266 | 0.726 | -0.2223 | No |
| 46 | OR2C1 |  |  | 6325 | 0.709 | -0.2246 | No |
| 47 | KREMEN2 |  |  | 6329 | 0.708 | -0.2228 | No |
| 48 | A2BP1 |  |  | 6464 | 0.663 | -0.2311 | No |
| 49 | NTAN1 |  |  | 6465 | 0.663 | -0.2292 | No |
| 50 | NOMO3 |  |  | 6474 | 0.659 | -0.2279 | No |
| 51 | SEC14L5 |  |  | 6552 | 0.633 | -0.2319 | No |
| 52 | TNP2 |  |  | 6696 | 0.588 | -0.2411 | No |
| 53 | TFAP4 |  |  | 6777 | 0.561 | -0.2455 | No |
| 54 | PKD1 |  |  | 6888 | 0.522 | -0.2524 | No |
| 55 | ITGAL |  |  | 6992 | 0.490 | -0.2588 | No |
| 56 | OR1F2 |  |  | 6998 | 0.488 | -0.2578 | No |
| 57 | HBZ |  |  | 7026 | 0.477 | -0.2584 | No |
| 58 | UBE2I |  |  | 7108 | 0.449 | -0.2633 | No |
| 59 | TPSAB1 |  |  | 7157 | 0.434 | -0.2657 | No |
| 60 | PRM2 |  |  | 7335 | 0.370 | -0.2781 | No |
| 61 | TPSD1 |  |  | 7340 | 0.369 | -0.2773 | No |
| 62 | CDR2 |  |  | 7491 | 0.311 | -0.2878 | No |
| 63 | RAB11FIP3 |  |  | 7530 | 0.296 | -0.2898 | No |
| 64 | C16ORF35 |  |  | 7556 | 0.288 | -0.2909 | No |
| 65 | UBN1 |  |  | 7652 | 0.247 | -0.2974 | No |
| 66 | MAGMAS |  |  | 7727 | 0.221 | -0.3024 | No |
| 67 | ERCC4 |  |  | 7746 | 0.216 | -0.3032 | No |
| 68 | SSTR5 |  |  | 7818 | 0.181 | -0.3080 | No |
| 69 | CREBBP |  |  | 7868 | 0.158 | -0.3113 | No |
| 70 | HMOX2 |  |  | 7951 | 0.122 | -0.3172 | No |
| 71 | MYH11 |  |  | 7989 | 0.109 | -0.3197 | No |
| 72 | PDPK1 |  |  | 8096 | 0.072 | -0.3275 | No |
| 73 | OR1F1 |  |  | 8202 | 0.033 | -0.3354 | No |
| 74 | RSL1D1 |  |  | 8205 | 0.033 | -0.3355 | No |
| 75 | ARHGDIG |  |  | 8374 | -0.027 | -0.3482 | No |
| 76 | DNASE1 |  |  | 8375 | -0.027 | -0.3481 | No |
| 77 | TRAP1 |  |  | 8457 | -0.058 | -0.3541 | No |
| 78 | ZNF205 |  |  | 8481 | -0.070 | -0.3556 | No |
| 79 | SYNGR3 |  |  | 8579 | -0.116 | -0.3627 | No |
| 80 | LOC92017 |  |  | 8814 | -0.215 | -0.3799 | No |
| 81 | ZNF500 |  |  | 8832 | -0.225 | -0.3805 | No |
| 82 | SOLH |  |  | 8884 | -0.250 | -0.3837 | No |
| 83 | CACNG3 |  |  | 8940 | -0.282 | -0.3870 | No |
| 84 | CLCN7 |  |  | 8950 | -0.284 | -0.3869 | No |
| 85 | UMOD |  |  | 9039 | -0.328 | -0.3926 | No |
| 86 | FLJ11151 |  |  | 9079 | -0.350 | -0.3946 | No |
| 87 | KIAA0251 |  |  | 9290 | -0.451 | -0.4093 | No |
| 88 | THOC6 |  |  | 9404 | -0.513 | -0.4164 | No |
| 89 | BFAR |  |  | 9525 | -0.574 | -0.4238 | No |
| 90 | IGFALS |  |  | 9576 | -0.604 | -0.4259 | No |
| 91 | DNASE1L2 |  |  | 9765 | -0.704 | -0.4381 | No |
| 92 | GSPT1 |  |  | 9841 | -0.751 | -0.4417 | No |
| 93 | ZNF174 |  |  | 10088 | -0.901 | -0.4578 | No |
| 94 | ADCY9 |  |  | 10125 | -0.918 | -0.4578 | No |
| 95 | PMM2 |  |  | 10229 | -0.977 | -0.4629 | No |
| 96 | TSC2 |  |  | 10272 | -1.004 | -0.4631 | No |
| 97 | PRSS22 |  |  | 10293 | -1.026 | -0.4617 | No |
| 98 | CORO7 |  |  | 10294 | -1.027 | -0.4587 | No |
| 99 | C16ORF51 |  |  | 10330 | -1.050 | -0.4583 | No |
| 100 | DEXI |  |  | 10461 | -1.130 | -0.4649 | No |
| 101 | USP7 |  |  | 10507 | -1.169 | -0.4650 | No |
| 102 | GNG13 |  |  | 10545 | -1.202 | -0.4643 | No |
| 103 | ABCC6 |  |  | 10746 | -1.350 | -0.4756 | Yes |
| 104 | SRRM2 |  |  | 10750 | -1.354 | -0.4719 | Yes |
| 105 | LOC399491 |  |  | 10818 | -1.403 | -0.4729 | Yes |
| 106 | C16ORF34 |  |  | 10823 | -1.408 | -0.4692 | Yes |
| 107 | KIAA0350 |  |  | 10831 | -1.413 | -0.4656 | Yes |
| 108 | NPIP |  |  | 10855 | -1.433 | -0.4632 | Yes |
| 109 | HBA2 |  |  | 10898 | -1.473 | -0.4621 | Yes |
| 110 | C16ORF28 |  |  | 10913 | -1.488 | -0.4589 | Yes |
| 111 | FLJ42627 |  |  | 10940 | -1.518 | -0.4565 | Yes |
| 112 | DNAJA3 |  |  | 11017 | -1.580 | -0.4577 | Yes |
| 113 | CLUAP1 |  |  | 11071 | -1.636 | -0.4570 | Yes |
| 114 | MGC3771 |  |  | 11134 | -1.690 | -0.4568 | Yes |
| 115 | LUC7L |  |  | 11205 | -1.747 | -0.4570 | Yes |
| 116 | NARFL |  |  | 11228 | -1.766 | -0.4536 | Yes |
| 117 | MGRN1 |  |  | 11330 | -1.854 | -0.4559 | Yes |
| 118 | HBA1 |  |  | 11398 | -1.934 | -0.4554 | Yes |
| 119 | SEPX1 |  |  | 11416 | -1.948 | -0.4510 | Yes |
| 120 | MRPL28 |  |  | 11442 | -1.980 | -0.4472 | Yes |
| 121 | C16ORF72 |  |  | 11456 | -1.995 | -0.4424 | Yes |
| 122 | TBL3 |  |  | 11466 | -2.002 | -0.4373 | Yes |
| 123 | NAGPA |  |  | 11522 | -2.053 | -0.4355 | Yes |
| 124 | C16ORF68 |  |  | 11546 | -2.084 | -0.4312 | Yes |
| 125 | N-PAC |  |  | 11552 | -2.088 | -0.4256 | Yes |
| 126 | GBL |  |  | 11584 | -2.110 | -0.4218 | Yes |
| 127 | ZC3H7A |  |  | 11618 | -2.140 | -0.4181 | Yes |
| 128 | DECR2 |  |  | 11716 | -2.238 | -0.4190 | Yes |
| 129 | TPSG1 |  |  | 11848 | -2.368 | -0.4221 | Yes |
| 130 | NTHL1 |  |  | 11885 | -2.421 | -0.4178 | Yes |
| 131 | TMEM8 |  |  | 11990 | -2.547 | -0.4184 | Yes |
| 132 | THUMPD1 |  |  | 12075 | -2.664 | -0.4170 | Yes |
| 133 | RHOT2 |  |  | 12104 | -2.709 | -0.4113 | Yes |
| 134 | RGS11 |  |  | 12128 | -2.744 | -0.4051 | Yes |
| 135 | SPSB3 |  |  | 12145 | -2.762 | -0.3983 | Yes |
| 136 | PARN |  |  | 12302 | -2.967 | -0.4016 | Yes |
| 137 | TMC5 |  |  | 12334 | -3.001 | -0.3952 | Yes |
| 138 | KIAA0430 |  |  | 12380 | -3.066 | -0.3898 | Yes |
| 139 | RHBDL1 |  |  | 12412 | -3.117 | -0.3831 | Yes |
| 140 | NUBP2 |  |  | 12418 | -3.132 | -0.3744 | Yes |
| 141 | TELO2 |  |  | 12425 | -3.144 | -0.3658 | Yes |
| 142 | ATP6V0C |  |  | 12506 | -3.271 | -0.3624 | Yes |
| 143 | NDE1 |  |  | 12586 | -3.408 | -0.3585 | Yes |
| 144 | RAB40C |  |  | 12596 | -3.428 | -0.3493 | Yes |
| 145 | PIGQ |  |  | 12611 | -3.451 | -0.3403 | Yes |
| 146 | BAIAP3 |  |  | 12628 | -3.484 | -0.3314 | Yes |
| 147 | FLJ14154 |  |  | 12668 | -3.553 | -0.3241 | Yes |
| 148 | SLC9A3R2 |  |  | 12716 | -3.649 | -0.3171 | Yes |
| 149 | C16ORF33 |  |  | 12722 | -3.662 | -0.3069 | Yes |
| 150 | C16ORF30 |  |  | 12754 | -3.754 | -0.2984 | Yes |
| 151 | PLA2G10 |  |  | 12843 | -3.988 | -0.2935 | Yes |
| 152 | BCKDK |  |  | 12859 | -4.025 | -0.2830 | Yes |
| 153 | ZNF263 |  |  | 12864 | -4.047 | -0.2715 | Yes |
| 154 | NME4 |  |  | 12911 | -4.183 | -0.2629 | Yes |
| 155 | HAGH |  |  | 12925 | -4.250 | -0.2516 | Yes |
| 156 | MPG |  |  | 12973 | -4.396 | -0.2424 | Yes |
| 157 | RAB26 |  |  | 12975 | -4.402 | -0.2297 | Yes |
| 158 | E4F1 |  |  | 12980 | -4.414 | -0.2173 | Yes |
| 159 | MRPS34 |  |  | 12990 | -4.433 | -0.2051 | Yes |
| 160 | POLR3K |  |  | 12995 | -4.441 | -0.1925 | Yes |
| 161 | HCFC1R1 |  |  | 12997 | -4.445 | -0.1797 | Yes |
| 162 | ABCA3 |  |  | 13025 | -4.531 | -0.1686 | Yes |
| 163 | ABAT |  |  | 13071 | -4.721 | -0.1584 | Yes |
| 164 | IFT140 |  |  | 13109 | -4.861 | -0.1471 | Yes |
| 165 | MKL2 |  |  | 13142 | -5.001 | -0.1351 | Yes |
| 166 | CACNA1H |  |  | 13148 | -5.021 | -0.1209 | Yes |
| 167 | DCI |  |  | 13177 | -5.217 | -0.1079 | Yes |
| 168 | METRN |  |  | 13204 | -5.403 | -0.0942 | Yes |
| 169 | STUB1 |  |  | 13220 | -5.581 | -0.0792 | Yes |
| 170 | ROGDI |  |  | 13221 | -5.582 | -0.0630 | Yes |
| 171 | RHBDF1 |  |  | 13243 | -5.792 | -0.0478 | Yes |
| 172 | C16ORF45 |  |  | 13257 | -6.044 | -0.0313 | Yes |
| 173 | C16ORF24 |  |  | 13267 | -6.176 | -0.0141 | Yes |
| 174 | EMP2 |  |  | 13273 | -6.228 | 0.0036 | Yes |
Table: GSEA details [plain text format]

  

Fig 2: CHR16P13: Random ES distribution      
 Gene set null distribution of ES for **CHR16P13**
